# Co‐creating an everyday language illustration of learning health systems alongside patient, caregiver, and community partners

**DOI:** 10.1002/lrh2.70033

**Published:** 2025-09-08

**Authors:** Shelley Vanderhout, Maureen Smith, Nakia Lee‐Foon, Amanda Doherty‐Kirby, Rona Fleming, Don Grant, Annie‐Danielle Grenier, John Grogan, Roger Farley, Margaret King, Chris Johnston, Virgil Luca, Lisa Ridgway, Donna Rubenstein, Candace Skrapek, Kerry Kuluski

**Affiliations:** ^1^ Institute for Better Health Trillium Health Partners Mississauga Ontario Canada; ^2^ Institute of Health Policy, Management, and Evaluation University of Toronto Toronto Ontario Canada; ^3^ Patient, Caregiver, or Community Partner

**Keywords:** accessibility, co‐design, engagement, patient partnership

## Abstract

**Introduction:**

Patients, caregivers, and community partners (PCC) can have a variety of roles in learning health systems (LHS), such as contributing their data from healthcare encounters to embedded, continuous engagement where they identify health system priorities, guide operational, research, and quality improvement decisions, and facilitate knowledge sharing and implementation. Despite many LHS models placing emphasis on PCC, little has been done to help members of the public understand what a LHS is or initiate dialogue about how they can learn more and become engaged. We brought together a national network of PCC to co‐create an everyday language, arts‐based resource for the public to learn what a LHS is and how it relates to patient care journeys.

**Methods:**

Thirteen PCC with LHS experience from across Canada attended two 2‐h virtual workshops to generate ideas on how to better define LHS using everyday language, determine accessible ways to share this information, and co‐design a comic strip that can be widely shared across diverse settings and communities.

**Results:**

We co‐created a six‐panel comic strip that depicts a relatable patient experience of waiting in an emergency department. The comic shows that in a LHS, patients are invited to contribute their perspectives about improving healthcare and support implementing and testing new ideas in clinical settings. Creating this comic was considered important for various reasons: to promote a common language around LHS, to build trust between health systems and the public, and to widen the community of PCC who are engaged in LHS activities.

**Conclusions:**

This comic is intended to build capacity for LHS culture, where the public can understand how continuous learning and improvement fit within health care, and learn about opportunities for engagement in LHS.

## 1 INTRODUCTION

A learning health system (LHS) aims to embed research and quality improvement (QI) “learning cycles” into everyday health care that combine co‐design with patients, caregivers, and community partners (PCC), population health analytics, real‐time evidence synthesis, implementation, and evaluation to drive continuous innovation and improvements in care.^1^, ^2^ A central feature of LHS is intended to be embedded, continuous engagement of PCC to identify learning priorities, guide operational, research, and QI decisions, and facilitate knowledge sharing and implementation. In practice, though, PCC engagement is not always the core driving mechanism of LHS^3^, ^4^ and dialogue about priorities and decision making largely takes place among researchers and health system leaders rather than with PCC.^5^

The concept of a LHS remains foreign to most members of the public for several reasons: it is a relatively new model of bridging academic research and healthcare; there is variable language used to define and describe LHS, even among experts; and across diverse health system contexts, LHS take shape differently. This presents a challenge for PCC to learn about LHS, understand their possible roles as part of a LHS, and pursue opportunities to engage in priority setting, co‐design, and knowledge translation.

There is a need for accessible and relevant resources that can be easily shared with and understood by the public to initiate conversations that define the LHS, what it means to partner, and how to increase LHS involvement if desired. Despite their appetite to be included in shaping ideal processes and outcomes of engagement,^5^ to our knowledge, PCC have not yet been invited to co‐produce public‐friendly, accessible resources about the concept of a LHS. In this article, we describe how researchers and PCC collaborated to produce an everyday language, arts‐based resource for the general public to learn what a LHS is and how it threads throughout patient care journeys.

## 2 METHODS

Through our existing networks and leaders of provincial Strategy for Patient‐Oriented Research (SPOR) Support Units, we invited PCC from across Canada to attend two, 2‐h virtual workshops in September and November 2024. We aimed to include PCC who represented a breadth of geography, experiences with LHS, and different communities such as rural, urban, Indigenous, and Francophone. To this end, participants were encouraged to draw upon both their lived experiences, when possible, as patients or PCC in a LHS, and their ideas, visions, or hopes for how a LHS could take shape. The workshops were designed and led by two researchers (KK, SV) and a PCC (MS). We recorded the sessions, generated transcripts, and took detailed notes during each to capture key points and perspectives. Prior to the workshops, we provided participants with foundational resources about LHS to help prepare for discussion, and afterwards, a $200 gift card as an honorarium for participating in both sessions. Following each session, meeting notes and transcripts were summarized by SV, and KK and MS reviewed and verified key concepts.

In the first workshop, we introduced the concept of a LHS to provide common understanding and consistent terminology, and held a discussion to reach consensus on the vision and target audience for the resource we would co‐design in the workshops. We asked participants to describe what a LHS meant to them and why a public‐friendly LHS resource could be important. Participants considered different formats for the resource, such as an infographic, video, or comic strip, and decided that a comic strip could be an accessible, inviting, and easily shared modality. We brainstormed common patient experiences or journeys that could make a relevant and relatable vignette for the comic strip.

In the second workshop, we reviewed the first workshop's discussion, including ideas that were relevant but out of scope for this project and could be addressed in future work. Using the five gears of the LHS Action Framework^6^ (population health and analytics, evidence synthesis, patient and provider co‐design, implementation, and evaluation) as a guide, we envisioned six comic strip panels that illustrate a LHS and how it can weave into patient experiences. Finally, we discussed possible venues for sharing the comic strip, how it could spark discussion about LHS, and the intended impacts of producing and sharing this resource.

Following the second workshop, we partnered with a graphic designer to produce an initial draft, informed by notes from our workshops. The draft was shared with participants who suggested revisions; a final product was made. It was then translated into French by two workshop participants (MS, RF).

## 3 RESULTS

**FIGURE 1:**
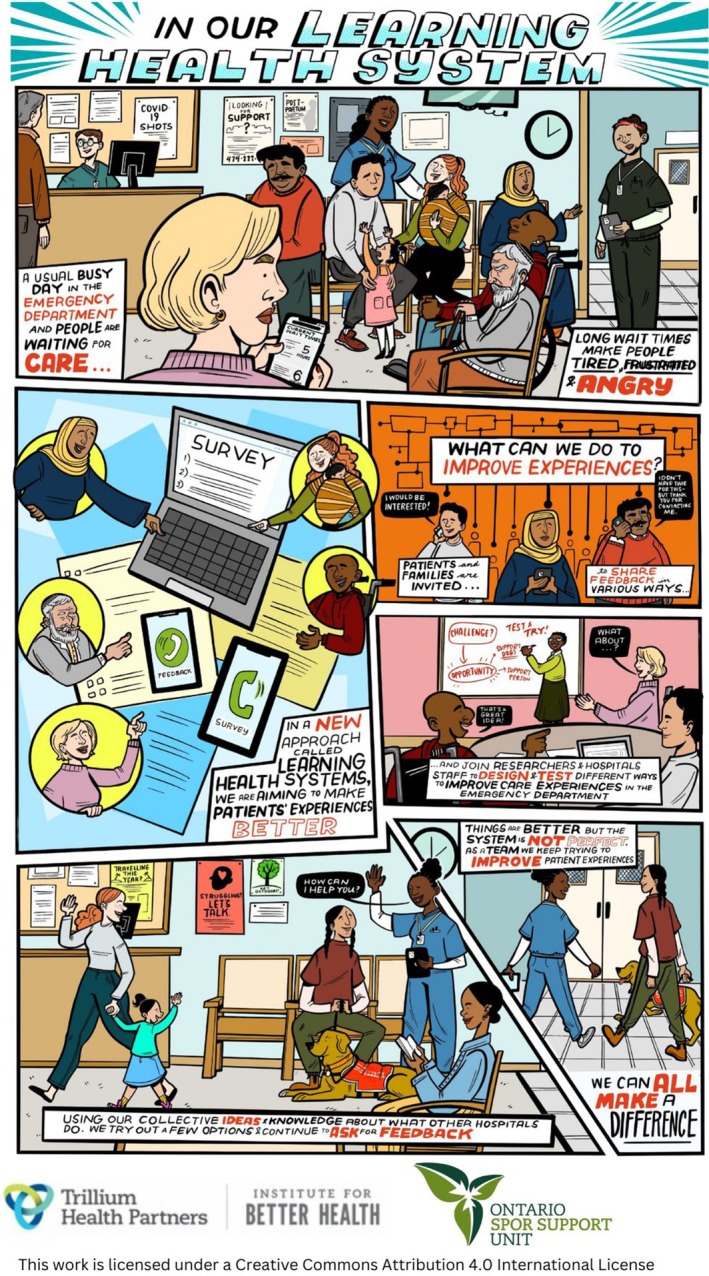
Learning health system comic strip.

Thirteen PCC from six provinces (Ontario, Quebec, British Columbia, Saskatchewan, Nova Scotia, and Prince Edward Island) participated in the co‐design workshops. The outcome of this collaboration is shown in Figure 1.

### 3.1 Our envisioned LHS

We agreed on a collective vision of a LHS: it is a health system with shared responsibility and accountability across patients, healthcare providers, researchers, and leaders. A LHS offers ongoing opportunities to PCC to share their experiences, expectations, and visions about healthcare, which can lead to multi‐perspective partnerships where people co‐design, implement, and evaluate ideas to improve care in research or QI projects. We agreed that a LHS is accountable to the community to share how patient data and feedback are used; outcomes of research and QI projects; and how learning will shape future improvements in care.

### 3.2 Purpose

We heard from participants that helping the public to learn about LHS was important for various reasons: to promote a common language around LHS; to build trust between health systems and the public, especially where negative experiences in healthcare have occurred and reparation is needed; and to widen the community of PCC who are engaged in LHS activities such as co‐design, decision making, and knowledge translation to improve the diversity of views, priorities, and perspectives brought to those discussions. Participants stressed that patients need to “see themselves” as vital parts of a LHS, and that their possible roles be presented in accessible, relevant, and approachable ways.

### 3.3 Setting

We decided that waiting for emergency department (ED) care is a common and relatable experience that can include anxiety, frustration, and exhaustion, and people are likely to have opinions (either good or bad) about how their encounter went. Some people may be willing to offer feedback while still present in the ED, while others may be overwhelmed or uninterested in dialogue in the midst of a stressful situation—both of which reflect realistic outcomes of invitations to engage in a LHS, and we wanted to capture that. Sometimes the ED is an “entry point” to a health system, where a patient or caregiver might be seeking care for the first time and is therefore impressionable to its culture, staff, and experience.

### 3.4 Narrative

Rather than focusing on one individual as the “main character” of the comic, which would be difficult for a wide range of audiences with varying ages, genders, and ethnicities to find relatable, we decided to depict several patients to allow people from many different walks of life to identify with the story as much as possible.

We thought the comic strip could present a crowded waiting room where patients show confusion, pain, frustration, and concern, but there is a team asking for patients and caregivers to share feedback about their experiences in surveys and interviews. People who provide this feedback notice that the team actually cares about their perspectives and intends to act on what they hear, and invite patients and caregivers to be part of a group of researchers and health care providers to co‐design new ways to improve ED experiences. Some patients choose to engage further with this group, while others opt not to participate but still would like to stay informed about their work. Together, the group reviews different types of evidence such as existing research, approaches that other hospitals have taken to improve ED experiences, and local patient feedback. They work collaboratively to create and test ideas that might improve experiences in their setting, such as therapy dogs, having designated support staff in the ED, and connecting patients with appropriate care providers after they go home, or digital tools to triage patients more effectively. In time, people see that their feedback and some of these new ideas are implemented, and the team measures their impact and makes improvements. Outcomes and results of the new interventions the team introduced are shared back with the health system community through social media, hospital newsletters, and posters, and everyone is invited to contribute to further iterations. The system is not perfect, but better than before, and is on a path to continuous improvement that incorporates a range of perspectives and types of evidence.

### 3.5 Intended Impacts

A significant portion of workshop dialogue centered on participants' visions for the comic's potential impact. They wanted readers to come away with an awareness that they have a critical role in shaping healthcare improvement by learning together, encouragement to share their perspectives and experiences, and an understanding of the importance and potential impact of doing so. Ultimately, if people learn that LHS value and act on what they hear from PCC voices, the public could increase their trust and confidence in health systems.

Participants recognized that this comic strip would not provide a detailed nor technical description of what a LHS is and how it functions across different settings, but rather would depict one in a way that resonates across common patient experiences, and could prompt conversation and curiosity to learn more. For example, readers may wonder about why they might choose to participate in LHS activities, how to find out more about engagement opportunities, and where they might be able to share their experiences and priorities. We agreed that by design, the comic strip will be a conversation starter and is intended to be shared in settings where dialogue can take place. For example, in hospital or community Patient and Family Advisory Council meetings where researchers and clinicians engage with PCC, hospital town hall events where the public is invited to share dialogue about priorities and experiences, or bulletin boards in hospitals, primary care offices, and public health and community centers where staff are available to answer questions and provide more information.

## 4 FUTURE DIRECTIONS

Throughout our discussions, participants shared a wealth of ideas that were out of scope for this project but a springboard for future work, such as helping the public understand the range of their possible roles in a LHS: a “data donor” (which means they contribute their anonymized medical record information for research and QI), taking part in priority‐setting discussions about future research or QI projects, or co‐designing a new model of care. We discussed other settings where a LHS could be illustrated, such as primary care, that could guide future dialogue about how a LHS is relevant to other patient and caregiver journeys. Participants also identified that LHS often need to creatively broaden their PCC engagement approaches to capture a wider range of perspectives and experiences, such as through the Patient Advisory Network (patientadvisors.ca) or Patient Voices British Columbia (patientvoicesbc.ca).

## 5 DISCUSSION

As a LHS community, we are learning how to support dialogue between LHS and PCC to understand their needs, experiences, and preferences for how they want to be involved in priority‐setting, conducting research and QI, and mobilizing knowledge. We collaborated to illustrate a LHS in a way that resonates with common healthcare experiences and identify accessible ways for PCC to participate in LHS, such as donating their anonymized healthcare data, providing feedback in surveys and interviews, and joining a collaborative team to improve patient safety, care delivery, and experiences through research and quality improvement. Though patients and the public have not been provided many opportunities to share this dialogue, participants in our workshops articulated a clear vision of a LHS: a system that offers opportunities for PCC to share their experiences, expectations, and visions with healthcare providers, researchers, and leaders, and that is accountable to the community to share how patient data and feedback are used, outcomes of research and QI projects, and how learning will shape future improvements in care. We intentionally designed this resource to spark dialogue and invoke curiosity among PCC and health system partners to co‐envision how a patient‐centered LHS can take shape across different contexts.

Our comic strip fills a gap in what is available to support public awareness and learning about LHS and initiate dialogue about how health systems can make space for community voices, priorities, and experiences at the most basic level. To meet more advanced learning needs, there are complementary resources available; for example, the Centre of Excellence on Partnership with Patients and the Public and the Unité de soutien SSA Québec partnered with PCC to co‐produce a roadmap for PCC partnership in LHS, which clearly explains roles, winning conditions, and potential challenges across LHS learning cycle stages.^7^ For even deeper learning, the McMaster Health Forum and Ontario SPOR Support Unit offer a 90‐min online LHS “nano‐course” (ossu.ca/resources/master‐class), co‐delivered by health system and patient partners and oriented around embedding PCC in LHS co‐design and operations.

Our approach had several strengths. Workshop participants fostered a strong sense of collaboration and validated and enhanced our understanding of lived experience in a LHS. In the spirit of empowering PCC and including them in leadership and decision‐making in a LHS, we intentionally combined health system and patient perspectives in the design and oversight of this work. We were able to include a range of PCC from across Canada, who represent a variety of perspectives, interactions with different health systems and providers, and knowledge about and experience with a LHS. This also brought forward firsthand views of what information resonates and where and how it should be shared, which we hope will maximize the impact of our work.

We acknowledge some limitations to our approach. While we made efforts to ensure our participants represented different backgrounds, in future work we will recruit PCC more systematically to capture all provinces and territories in Canada and greater age and cultural diversity. Though it was useful for us to include people who were familiar with LHS, including those who are newer to the concept of LHS in future work will broaden and diversify our dialogue with members of the public. The virtual format of our workshops promoted accessibility for people from across Canada to participate; however, access to technology and digital literacy may have presented barriers for others. While we received informal positive feedback from PCC about their experiences of participating in these workshops and enthusiasm to contribute to similar work in the future, we did not conduct a formal evaluation of the impacts of participation on their knowledge about LHS.

To assess the impact of our work, next steps will include monitoring knowledge mobilization metrics such as web page views where the comic is posted online and conference and webinar presentations that include the comic, and an online engagement survey embedded within the comic to assess its effectiveness in increasing knowledge about LHS among both English and Francophone audiences. In response to participants' eagerness to contribute to similar work in the future and the wealth of ideas brought to these workshops that were relevant but considered out of scope, next steps may also include co‐creating other resources that present vignettes of different engagement styles or clinical contexts; impacts of these could be compared with identify strengths and weaknesses of different approaches.

A LHS is intended to address population health needs, co‐design patient‐centered care, and create cultures of learning and continuous improvement where patients, healthcare providers, and researchers understand the value of their roles and contributions. However, there are few resources available to help the public understand what a LHS is, how it relates to their healthcare, and what it looks like to contribute to LHS activities such as research and QI projects, priority setting, and storytelling about their experiences, despite a clear desire to be included in this dialogue. This risks undermining the goal of a LHS to align priorities, decisions, and care innovations with patients' and communities' needs. Our co‐created comic strip can be used to build a LHS culture, where patients and the public can understand how continuous learning and improvement fit within healthcare and access opportunities for engagement in LHS.

## AUTHOR CONTRIBUTIONS

Shelley Vanderhout, Maureen Smith, Nakia Lee‐Foon, and Kerry Kuluski conceptualized and designed this project. Data collection was led by Shelley Vanderhout, Maureen Smith, and Kerry Kuluski, who also produced the first draft of the manuscript. Amanda Doherty‐Kirby, Rona Fleming, Don Grant, Annie‐Danielle Grenier, John Grogan, Roger Farley, Margaret King, Chris Johnston, Virgil Luca, Lisa Ridgway, Donna Rubenstein, Candace Skrapek, and Maureen Smith contributed their perspectives and lived experiences as data for this project. All authors reviewed and approved the manuscript as submitted.

## FUNDING INFORMATION

The funding for this work was provided by the Ontario Strategy for Patient Oriented Research (SPOR) Support Unit and Trillium Health Partners' Institute for Better Health.

## CONFLICT OF INTEREST STATEMENT

The authors declare no conflicts of interest.
